# Online Visibility and Scientific Relevance of Strabismus Research: Bibliometric Analysis

**DOI:** 10.2196/50698

**Published:** 2024-06-12

**Authors:** Aleksander Stupnicki, Basil Suresh, Saurabh Jain

**Affiliations:** 1 University College London Medical School London United Kingdom; 2 Royal Free London NHS Foundation Trust London United Kingdom

**Keywords:** strabismus research, squint, social media, scientific relevance, altmetrics, accuracy, medical knowledge, metric, bibliometric analysis, research, strabismus, online visibility, platform, evidence-based information, accessibility

## Abstract

**Background:**

Quality and accuracy of online scientific data are crucial, given that the internet and social media serve nowadays as primary sources of medical knowledge.

**Objective:**

This study aims to analyze the relationship between scientific relevance and online visibility of strabismus research to answer the following questions: (1) Are the most popular strabismus papers scientifically relevant? (2) Are the most high-impact strabismus studies shared enough online?

**Methods:**

The Altmetric Attention Score (AAS) was used as a proxy for online visibility, whereas citations and the journal’s impact factor (IF) served as a metric for scientific relevance. Using “strabismus” as a keyword, 100 papers with the highest AAS and 100 papers with the highest number of citations were identified. Statistical analyses, including the Spearman rank test, linear regression, and factor analysis, were performed to assess the relationship between AAS, citations, a journal’s IF, and mentions across 18 individual Web 2.0 platforms.

**Results:**

A weak, positive, statistically significant correlation was observed between normalized AAS and normalized citations (*P*<.001; *r*=0.27) for papers with high visibility. Only Twitter mentions and Mendeley readers correlated significantly with normalized citations (*P*=.02 and *P*<.001, respectively) and IF (*P*=.04 and *P*=.009, respectively), with Twitter being the strongest significant predictor of citation numbers (*r*=0.53). For high-impact papers, no correlation was found between normalized citations and normalized AAS (*P*=.12) or the IF of the journal (*P*=.55).

**Conclusions:**

While clinical relevance influences online attention, most high-impact research related to strabismus is not sufficiently shared on the web. Therefore, researchers should make a greater effort to share high-impact papers related to strabismus on online media platforms to improve accessibility and quality of evidence-based knowledge for patients.

## Introduction

Patients, health care professionals, and researchers increasingly use social media and online platforms as a source of knowledge, health care news, and scientific research [1]. Despite the worldwide prevalence of strabismus remaining stable at around 2% [2], the public’s online interest in the topic has been rising, a trend reflected by the increasing popularity of queries related to the disease over the past 2 decades according to Google Trends. Due to this increasing reliance on online platforms, it is essential to ensure the quality and relevance of scientific data that are commonly accessed on the web, especially for lay members of the public who may lack the skills or time to assess that themselves.

To quantify the relevance of research within the field of medical science, the number of citations and impact factor (IF) of the journal are used most frequently [3]. The dissemination of the same academic information through platforms used by the general public, on the other hand, can be most reliably quantified by the Altmetric Attention Score (AAS), a real-time weighted measure of mentions across all Web 2.0 social media platforms [4,5].

Bibliometric analyses using altmetrics and other scientometrics have been conducted previously in the field of ophthalmic research to evaluate publication trends [6], disruptiveness of papers [7], or research productivity [8]. To date, however, there have been no such analyses within the subspecialty of strabismus, despite the pervasiveness of the disease.

Therefore, we decided to analyze the relationship between the scientific relevance of strabismus research and its contributions to the online sphere, in order to answer the following questions: (1) Are the most popular strabismus papers scientifically relevant? (2) Are the most high-impact strabismus studies shared online enough?

## Methods

In line with the best practice of literature searching [9], a thesaurus synonym search was performed to identify appropriate keywords for database search. As of January 2023, the thesaurus does not identify synonyms for “strabismus,” and the *Cambridge Dictionary* confirms it is the only medical term to describe the condition [10]. No morphological variation of the term has been identified, eliminating the need for the use of truncation in keyword searches.

Therefore, a list of research papers including the keyword “strabismus” was generated on January 27, 2023, with Altmetric Explorer with no other restrictions (search period: January 2011 to January 2023). The keyword search engine in Altmetric Explorer yields comprehensive results including outputs that match the keyword across publication title, author name, or journal title [11]. Hence, the pooled list was then filtered by a consultant ophthalmologist according to relevance to include 100 papers with the highest AAS (a total of 255 titles and abstracts were analyzed to compile 100 relevant publications). Additional preliminary searches using lay synonyms of strabismus, including “squint” and “cross-eye,” were performed but yielded no relevant or sufficiently high AAS results for inclusion, proving the keyword “strabismus” captures the bona fide core of publications in the field.

On the same day (January 27, 2023), for each of the papers, Web of Science (WoS) was used to add information on the number of citations, time since publication, and IF of the journal at the time of publication; other metrics traditionally used to assess the quality and relevance of scientific research [12]. Additional data on the source of AAS, including mentions across (1) news, (2) blogs, (3) Twitter, (4) peer review, (5) Facebook, (6) Wikipedia, (7) LinkedIn, (8) Weibo, (9) Google+, (10) Reddit, (11) Pinterest, (12) F100, (13) Q&A, (14) policy, (15) patent, (16) video, (17) syllabi, and (18) Mendeley, were pooled from Altmetric website and evaluated to characterize the field.

For systematic comparison, the same approach to searching was implemented to yield a list of papers with the highest number of citations: on January 27, 2023, WoS was used to generate a list of 100 papers including the keyword “strabismus” with the highest number of citations, excluding papers published before 2011, and the year Altmetric Explorer was founded and started tracking the AAS (search period: January 2011 to January 2023). No other filters were applied to the search. On the same day, the AAS for each of the papers was manually pooled from Altmetric website. Data on time since publication and the journal’s IF at the time of publication were extracted from WoS. To account for temporal differences [13], the values for AAS and citations for both groups have been then normalized per year since publication.

Kolmogorov-Smirnov test was used to verify that the distribution of the data does not follow a normal distribution, and Spearman rank correlation coefficient was used to test for correlation between all variables. Following correlational calculations, linear regression analysis and factor analysis were performed to explain patterns among correlated variables, both of which are statistical techniques commonly used in altmetric research [14,15]. SPSS (IBM Corp) was used for all statistical calculations. Statistical significance was defined as *P*<.05.

### Ethical Considerations

No ethics board approval was required, as the study did not involve any human participants.

## Results

### Correlation Analysis

The normalized AASs of the 100 papers with the highest online visibility (median AAS 11, IQR 6-16) correlated significantly with normalized citations (*P*<.001) but demonstrated a weak strength of the relationship (*r*=0.27) for papers with AAS <150. To achieve this result, we excluded 3 outlier papers with significantly higher AASs (922, 413, and 169, respectively, compared to median 11, IQR 6-16; *z* score>3), which would otherwise skew the statistical analysis. Spearman rank test demonstrated no correlation between the normalized AAS and the IF of the journal (*P*=.15) or time (*P*=.37).

For the 100 papers with the highest number of citations (median 30, IQR 15-45), no statistically significant correlation was found between normalized citations and the normalized AAS (*P*=.12) or IF of the journal (*P*=.55), but as expected, they correlated significantly with time (*P*=.01).

Upon analysis of AAS sources, we found a weak, positive, statistically significant correlation between normalized citations and Twitter mentions (*P*=.02; *r*=0.27), normalized citations and Mendeley readers (*P*<.001; *r*=0.40), and normalized citations and policy mentions (*P*=.02; *r*=0.24) for the 100 papers with highest AAS. The same variables showed a weak, positive, statistically significant correlation with the IF of the journal at the time of publication: Twitter and IF (*P*=.04; *r*=0.25), Mendeley readers and IF (*P*=.009; *r*=0.32), and policy mentions and IF (*P*=.04; *r*=0.26).

Correlations between the number of mentions in the news, on blogs, in peer-reviews, on Facebook, Wikipedia, LinkedIn, Reddit, Google+, Weibo, Pinterest, syllabi, or video and normalized citations or IF were not significant at *P*<.05 (Multimedia Appendix 1).

### Multivariate Analysis

To better understand variance among the correlated variables, a linear regression model was run with normalized citations as the dependent variable and Twitter mentions, Mendeley readers, and policy mentions as covariates. ANOVA test showed significant variance within the sample, confirming the suitability of the test (*P*<.001). We obtained an *R*^2^ value of 0.31, indicating that 31% of the variance within citations can be explained cumulatively by the 3 AAS sources. Only Twitter and policy mentions, however, were significant predictors (*P*<.001 and *P*=.004 respectively), with Twitter mentions being the most important predictor as indicated by the highest standardized coefficient (*r*=0.53).

### Factor Analysis

Factor analysis was performed on metrics with adequate data for the papers with the top 100 AAS normalized citations, normalized AAS, IF, time since publication (months), Twitter mentions, Mendeley readers, and policy documents. Bartlett test of sphericity indicated an approximate chi-square value of *χ*^2^_93_=124.4 (*P*<.001) and a Kaiser-Meyer-Olkin adequacy value of 0.607, together indicating the suitability of the data set for factor analysis. Three factors were identified across these variables: factor 1 between Twitter, Mendeley, AAS, and citations; factor 2 between Mendeley, policy, and citations; and factor 3 between IF and time.

## Discussion

### Principal Findings

The significant, yet, weak correlation of AAS with citations for papers with the highest online visibility shows that the clinical relevance of strabismus-related publications (as measured by citations) can contribute to increased online popularity but is not the sole determining factor. Furthermore, the lack of correlation between AAS and IF demonstrates that the relative importance of a journal in the field (and consequently the paper) does not determine its online popularity, which raises questions about the quality of strabismus research receiving the most online attention.

Through correlational and multivariate analysis of mentions across individual Web 2.0 platforms, we have demonstrated Twitter mentions to be a significant and strong predictor of citations for the most popular strabismus papers. Although prior studies demonstrated a more significant, causative impact of tweets on citation numbers [16], the relationship can differ between fields [17] and seems to be statistically significant for strabismus research, albeit moderate compared with other research domains. Overall, our findings imply that dissemination of strabismus research through Twitter can have an impact on scholarly visibility and subsequently citation rates.

Furthermore, we demonstrated a lack of statistically significant correlations between traditional scientometrics (citations and IF) and mentions across other social or media platforms, which has been also observed in other fields of research [17]. This reveals existing gaps that require more references to research papers on strabismus, including social media platforms like Facebook or LinkedIn, as well as critical, knowledge-oriented pages such as Wikipedia.

Furthermore, in terms of factor analysis, factor 1 linking AAS, citations, Mendeley, and Twitter likely suggests that for strabismus research, there is a degree of overlap regarding the user bases or networks between Mendeley and Twitter, despite the former being considered a platform largely used by academic professionals as opposed to the more widely public microblogging service [18]. It may also indicate that highly cited papers are receiving engagement and being discussed in both academic and general public networks indicating that such papers may have a wider social impact. Factor 2 linking Mendeley, policy documents, and citations may suggest that for highly cited papers, there is increased interest and readership on Mendeley—a proportion of which may be faculty and departmental figures. This in turn may lead to policy mentions for impactful research papers. Therefore, this suggests that papers with high citation counts and academic impact may be influencing policies and organizational standards [19]. Factor 3 linking IF and time could be due to the overall increase and growth of the cited strabismus literature over time; however, this is less relevant to our research question.

For the 100 papers with the highest number of citations, the lack of correlation between citations or IF and the AAS suggests that clinical relevance or perceived prestige related to the publishing journal does not affect the online visibility of strabismus papers. Researchers publishing in the strabismus realm should, therefore, make a greater effort to share their high-impact papers on social media. In turn, this could increase the visibility and accessibility of their research, especially for the lay public who rarely browses journals for medical knowledge, enhance collaboration, and further enhance the overall impact of their research.

### Strengths and Limitations

AAS itself is a useful tool for authors to get quick, up-to-date insight into the performance of their papers on the web. It is crucial, however, to bear in mind the inherent limitations of AAS due to the fact that it is ultimately only a metric of “mentions” or “posts” and is not an indicator of research quality or legitimacy [20]. In isolation, it may be deemed unreliable, as “viral” papers that do not exhibit robust research methodology or present sensible conclusions may still acquire a high AAS. Furthermore, the AAS does not account for following or website traffic, therefore providing no information on the actual number of viewers. As a result, a frequently mentioned paper can effectively have low visibility and reception, despite a high AAS. Although Twitter mentions and Mendeley readership seem to have some impact on citation numbers of the most popular strabismus papers, they only account for a small proportion of the variance within citation data (31%). Thus, using alternative metrics, like tweets, as predictors of scientific contribution and success does not constitute a comprehensive and precise appraisal method, as demonstrated before across several other fields [16,17].

Furthermore, citations take a longer time to accumulate, whereas AAS is updated almost in real time, so even when a new paper is published that can have high publicity in social media and among the scientific community, the citation numbers will lag months or sometimes years behind. Due to this phenomenon, there may be weaker or no association between citations and AAS for the latest research papers, which could skew the results of our 12-year view. Further statistical testing would be necessary to confirm that.

Additionally, in the case of strabismus research, both the AAS and citation numbers are characteristically low, which raises questions about the reliability of the data set, as it is analogical to having a small sample size. Various scientifically irrelevant factors can cause a high AAS, such as the topic of the paper, sensationalism, how easily it is understood by the general public, or the number of intersections of the topic with other branches of medicine. A good example of the effect of those confounding factors is a publication included in our data set entitled “Evidence That Leonardo da Vinci Had Strabismus,” which had the highest AAS of 922 (over 83 times the median score) but only 6 citations. This demonstrates that especially for papers with high AAS scores, the virality of the topic can have a higher impact on the AAS than its scientific significance.

### Conclusions and Future Directions

We have demonstrated that the clinical relevance of strabismus research contributes to the amount of online attention it receives. However, the most high-impact strabismus research is not sufficiently shared across online platforms. Therefore, we recommend that researchers make a greater effort to share high-impact studies on social media platforms to improve the quality of evidence-based information about strabismus and improve the accessibility of this knowledge. To maximize the societal impact of research, it is important to interact with both academic and general audiences, as shown by the overlap between Mendeley and Twitter engagement of strabismus publications.

Furthermore, we revealed Twitter mentions to be the strongest predictor of citation numbers for strabismus papers, highlighting the potential impact of social media on scholarly visibility. Our findings also highlight the need for engagement of strabismus researchers across a broader range of platforms, including Facebook, LinkedIn, or Wikipedia. However, due to its inherent biases and limitations, the AAS itself or mentions across specific platforms should only complement traditional metrics, such as IF and citations, to provide a broader picture of the publicity of the paper but should not act as a stand-alone metrics for assessing the quality and relevance of strabismus papers.

## Appendix

Multimedia Appendix 1 Summary table of Spearman rank test for normalized citations, impact factor, and all 18 Web 2.0 platforms analyzed.

## Abbreviations

AAS: Altmetric Attention Score
IF: impact factor
WoS: Web of Science
